# Enhanced recovery of high-quality DNA from limited FFPE tissue for advancing cancer genomics

**DOI:** 10.1038/s41598-026-51594-9

**Published:** 2026-05-23

**Authors:** Shweta Singh, Sierra Vidaurri, Astrid Perez, Anupam Dhasmana, Swati Dhasmana, Monica M. Betancourt-Garcia, Murali Mohan Yallapu, Subhash C. Chauhan, Sheema Khan

**Affiliations:** 1 Division of Cancer Immunology and Microbiology, ISU Medicine and Oncology, School of Medicine, The University of Texas Rio Grande Valley, McAllen, TX 78504 USA; 2 South Texas Center of Excellence in Cancer Research, School of Medicine, The University of Texas Rio Grande Valley, McAllen, TX 78504 USA; 3 DHR Health Institute for Research and Development, Edinburg, TX USA

**Keywords:** FFPE tissue, DNA extraction, Sequencing quality, Cancer disparities, Genomic analysis, Biological techniques, Biotechnology, Cancer, Molecular biology

## Abstract

Archived formalin-fixed paraffin-embedded (FFPE) tissue samples are crucial assets in cancer research. However, despite the availability of commercial DNA purification kits, researchers face persistent challenges. The limited tissue availability, formalin-induced crosslinking, and DNA fragmentation in FFPE blocks often results in low DNA yield and compromised quality, hindering downstream applications. In this study, we optimized and standardized DNA purification methods to achieve high-yield, high-integrity DNA, making it ideal for DNA library preparation and high-throughput sequencing. Therefore, we optimized DNA extraction protocols using two Qiagen kits, the QIAamp DNA FFPE Tissue Kit and the QIAamp DNA FFPE Advanced Kit. The optimized methods significantly improved the yield and integrity of DNA isolated from limited FFPE tissue samples, with the advanced kit demonstrating superior performance and efficiency. This resulted in a two- to threefold increase in DNA concentration, as determined by spectrophotometric (NanoDrop) and fluorometric (Qubit) analyses. Gel electrophoresis and DNA integrity number (DIN) assessments confirm enhanced fragment preservation, suitable for downstream applications. Libraries prepared using DNA from the advanced protocol demonstrated higher sequencing quality and microbial diversity resolution. This study demonstrated that optimizing DNA purification methods for FFPE tissue samples can substantially improve the yield and integrity of DNA, paving the way for more accurate and comprehensive genomic analyses.

**Supplementary Information:**

The online version contains supplementary material available at 10.1038/s41598-026-51594-9.

## Introduction

Formalin-fixed paraffin-embedded (FFPE) tissue blocks are an invaluable resource in clinical diagnostics, cancer genomics, and translational biomedical research. Maintained in vast archives across institutions, these preserved samples represent an irreplaceable repository of patient-derived biological material, often linked with long-term clinical and pathological data. As such, FFPE tissues offer immense potential for retrospective molecular studies, biomarker discovery, and validation of disease-associated genetic and epigenetic alterations ^1,2^.

Despite their utility, FFPE tissues present substantial technical challenges, especially for nucleic acid-based applications. Formalin fixation introduces extensive cross-linking of nucleic acids and proteins, while paraffin embedding can entrap and degrade DNA. These processes often result in highly fragmented and chemically modified DNA, limiting the effectiveness of downstream molecular analyses such as polymerase chain reaction (PCR), genotyping, and next-generation sequencing (NGS). DNA isolated from FFPE tissues frequently suffers from low yield and compromised purity, with suboptimal A260/A280 and A260/A230 ratios, further complicating library preparation and sequence fidelity ^3–5^.

Given the importance of optimizing DNA recovery from FFPE tissues, several commercial kits have been developed to improve extraction outcomes. Among the most widely used methods are the Qiagen DNA FFPE Kit (standard kit) and the Qiagen DNA FFPE Advanced Kit (Advanced kit), both of which rely on silica-based column technology for DNA purification. While the standard kit is suitable for general-purpose extractions, the Advanced Kit incorporates enhanced chemistry to better accommodate degraded or limited input samples. Each kit includes standardized steps for deparaffinization, lysis, and protein digestion, but these protocols may still require further customization to maximize DNA yield, integrity, and downstream utility ^6^.

This study presents detailed methodological refinement and comparison of standard and advanced Qiagen FFPE DNA extraction kits, with a focus on improving DNA recovery for metagenomic and other high-sensitivity applications. We systematically optimized critical steps, including deparaffinization, ethanol washing, proteinase K digestion, and elution. Additionally, we explored the impact of homogenization and magnetic bead-based clean-up to improve DNA purity, especially in cases where the A260/A230 ratios were suboptimal. Our findings are particularly relevant for laboratories working with limited or valuable clinical samples, where maximizing both the quantity and quality of extracted DNA is crucial for successful downstream analysis.

By integrating practical modifications into existing workflows, this study provides an improved and reproducible protocol for extracting high-quality DNA from FFPE tissues. These enhancements support more reliable performance in sensitive downstream applications such as metagenomic sequencing, mutation profiling, and epigenetic analysis, making them valuable contributors to molecular pathology and translational research.

## Material and methods

### Sample collection

Archival FFPE blocks containing liver carcinoma tissue samples were obtained from the South Texas Center of Excellence in Cancer Research (STCECR) biobank, sourced from Doctors Hospital at Renaissance (DHR), for DNA extraction. All analyzed samples were obtained during routine diagnostic procedures. Informed consent was obtained from all human participants and/or their legal guardians prior to inclusion in the study. To maintain patient confidentiality, all personal identifiers were anonymized, and no identifiable information was accessible throughout the study. The IRB protocol and guidelines were approved by the Institutional Review Board (IRB) UTRGV. A total of 60 FFPE tissues were taken for DNA extraction. FFPE blocks were used to obtain 10 μm tissue sections via the Microtome facility at the Integrated Cancer Research Core of the South Texas Center of Excellence in Cancer Research (STCECR), UTRGV. Prior to the implementation of a specific procedure, all the samples were treated equally. All methods were carried out in accordance with relevant guidelines and regulations.

### Tissue sectioning and processing

The tissue sections were cut from FFPE blocks using a standard rotary microtome (Epredia HM 355S Automatic Microtome) ^7^. We optimized our DNA isolation protocol using 4–6 FFPE tissue sections of 10 µm thickness per sample, and a total of 20 samples were included in this study. The sections analyzed were more than 10 years old, and the fixation dates for these samples are provided in the Supplementary Results (Supplementary Table S1). The approximate tissue surface area used for DNA extraction ranged from 5–30 mm^2^. Prior to DNA extraction, the paraffin surrounding the tissue core was carefully removed using a scalpel and tweezers, ensuring minimal disturbance to the tissue core while reducing potential paraffin contamination.

### Lab-standardized FFPE DNA protocol

Two commercially available kits for DNA extraction from FFPE tissue samples were compared: the QIAamp DNA FFPE Tissue Kit (Standard Kit; Qiagen, Cat. No. 56404) and the QIAamp DNA FFPE Advanced Kit (Advanced Kit; Qiagen, Cat. No. 56604). We evaluated these kits based on extraction time, yield, and quality. All procedures were performed following the manufacturer’s protocols, with modifications introduced to increase the DNA yield and quality. Specific adjustments, including DNA fragmentation and cross-linking, were made to address the unique challenges posed by FFPE samples.

The first DNA extraction procedure was performed using QIAamp DNA FFPE Tissue Kit. For simplicity, this kit is referred to as **“**Standard kit” throughout the manuscript. The Standard kit was used following the manufacturer’s instructions with specific modifications. Tissue sections of 5–10 μm thickness were prepared, and the first 2–3 sections were discarded if the sample surface had been exposed to air. The remaining sections were placed in a microcentrifuge tube and incubated at 56 °C for 1 h for baking. Samples were then subjected to 2–3 xylene washes (1 mL each) with centrifugation at 15,000 rpm for 2 min per wash to remove paraffin, followed by sequential ethanol washes at 100%, 75%, and 50% (1 mL each) with centrifugation at 15,000 rpm for 3–5 min per wash. The tissue pellet was air-dried for 10 min and resuspended in 200 µL of Buffer ATL, then homogenized using a Fisherbrand™ 150 Homogenizer (Thermo Scientific, USA) for three cycles of 30 s each. Additional Buffer ATL was added as needed during homogenization, not exceeding 300 µL in total. 2.6 mg/ml of Proteinase K (40 µL of 20 mg/mL stock) was added, and samples were incubated overnight at 56 °C. The following morning, an additional 1.17 mg/ml of Proteinase K (20 µL of 20 mg/mL stock) was added, and incubation continued for 90 min. To reverse formaldehyde-induced crosslinks, samples were incubated at 90 °C for 1 h. After brief vortexing, samples were mixed with 200 µL Buffer AL and 200 µL 100% ethanol, vortexed again, and transferred to QIAamp MinElute columns for DNA purification. Columns were washed twice with 500 µL each of Buffers AW1 and AW2 by centrifugation at 8,000 rpm for 1 min, followed by a 100% ethanol wash. Columns were air-dried to remove residual ethanol, and DNA was finally eluted in Buffer ATE with an incubation step to maximize yield.

The second DNA extraction procedure utilized the QIAamp DNA FFPE Advanced Kit. For simplicity, this kit is referred to as “Advanced kit**”** throughout the manuscript. The Advanced kit was used following the manufacturer’s instructions with specific modifications to enhance DNA yield and integrity. FFPE tissue sections were incubated in a microcentrifuge tube at 56 °C for 1 h. Subsequently, 400 µL of Deparaffinization Solution (volume adjusted according to the number of scrolls) was added, followed by brief vortexing and incubation at 56 °C for 10 min. Samples were then cooled to room temperature. Homogenization was performed directly in the deparaffinization solution for 15–20 s using a Fisherbrand™ 150 Homogenizer (Thermo Scientific, USA). To the homogenized sample, 35 µL of Buffer FTB, 65 µL of RNase-free water, and 1.2 mg/ml Proteinase K (30 µL of 20 mg/mL stock) were added. The sample was further homogenized for 2–3 cycles of 15–20 s each. During this process, the Buffer FTB volume could be adjusted up to 40–50 µL, depending on tissue size and consistency. The mixture was then incubated overnight at 56 °C with shaking at 1000 rpm, followed by an additional incubation at 90 °C for 1 h without shaking to reverse formalin-induced crosslinks. After cooling to room temperature, the upper blue phase was carefully removed, and 150 µL of RNase-free water was added to the aqueous lysate. Subsequently, 2 µL of RNase A was introduced, and the sample was incubated for 2 min at room temperature. Next, 610 µg/ml of Proteinase K (20 µL of 20 mg/mL stock) was added, followed by incubation at 65 °C with shaking at 450 rpm for increased duration to 30 min. The lysate was then combined with 250 µL of Buffer AL and 100% ethanol before being transferred to a QIAamp UCP MinElute column. Columns were centrifuged at 15,000 × g for 30 s, followed by sequential washes with 500 µL each of Buffers AW1 and AW2 under the same centrifugation conditions. Two additional ethanol washes (250 µL each) were performed to ensure removal of residual salts and contaminants. Columns were air-dried to eliminate traces of ethanol before elution. DNA was eluted using 25–30 µL of ATE or TE buffer (pH 8.0). To maximize recovery, the eluate was reapplied to the column for a second elution.

### Assessment of the concentration, quality, and integrity of isolated DNA

The DNA concentration in the extracted samples was measured via two methods: spectrophotometry at 260 nm (NanoDrop 2000, Thermo Scientific) and fluorometry via the Qubit dsDNA Broad-Range Assay (Qubit 3.0 Fluorometer, Life Technologies, Invitrogen). DNA quality was assessed via the absorbance ratio at 260 nm and 280 nm (A260/A280). Samples with A260/A280 ratios between 1.8 and 2.0 were considered of acceptable quality. Additionally, Qubit fluorometric analysis was used to quantify the double-stranded DNA (dsDNA) content in each sample. A Qubit 1X dsDNA HS Assay Kit was used to measure the DNA concentration. This high-sensitivity assay can detect low concentrations of dsDNA, helping to conserve valuable samples. Quantification was performed using DNA standards of 0 ng/μl and 10 ng/μl, following the manufacturer’s instructions. DNA integrity was also assessed via the TapeStation system (4200 TapeStation System, Agilent), which requires only 2 µl of DNA sample and provides high-resolution DNA band visualization. This quality assessment can be further used for DNA sequencing and other downstream applications.

### Length-dependent PCR quality control

Length-dependent PCR quality control was performed to assess genomic DNA integrity by amplifying fragments of increasing length using conventional PCR (Bio-Rad). Each 50 µL reaction contained 2 × PCR Bestaq Mastermix (Cat. No. G464, Applied Biological Materials Inc.), 0.2 µM each of forward and reverse primers targeting *β-Actin* and *GAPDH*, and 100 ng of template DNA. Primers were designed using the PrimerQuest tool available on the Integrated DNA Technologies (IDT) website. Primer sets generating amplicons of varying lengths were selected, and their nucleotide sequences are provided in the Supplementary results (Supplementary Table S2). These primer sets were designed to evaluate the quality of extracted genomic DNA by assessing its ability to support amplification of progressively longer fragments PCR reactions were assembled on ice, briefly centrifuged, and subjected to an initial denaturation at 94 °C for 3 min, followed by 30–35 cycles of denaturation at 94 °C for 10 s, annealing at 51 °C for 30 s, and extension at 72 °C for 1 min. A final extension step was performed at 72 °C for 5 min. PCR products were resolved on 1.5% agarose gels prepared in 1 × TAE buffer and visualized under UV illumination. Successful amplification of longer *β-Actin* and *GAPDH* fragments was found as evidence of high-quality genomic DNA suitable for downstream sequencing applications.

### 16S rRNA gene-based metagenomic sequencing

16S metagenomic sequencing was performed using the Illumina MiSeq platform, following the Illumina MiSeq Library Preparation Guide (Illumina, USA) and a standardized protocol previously established in our laboratory ^8^. The hypervariable V3–V4 region of the 16S rRNA gene was amplified from genomic DNA using region-specific primers (341F/805R) containing Illumina adapter overhang sequences. Amplicons were purified via magnetic beads, and dual-index barcodes were added during a second Index PCR step. The final libraries were quantified, normalized, pooled, and sequenced on the Illumina MiSeq platform using paired-end 2 × 150 bp reads. The resulting FASTQ files were processed via the MOTHUR workflow on the Galaxy platform. Merged reads were clustered into operational taxonomic units (OTUs), followed by taxonomic classification via the SILVA 16S rRNA gene database (version 138). Diversity and taxonomic analyses were then performed based on the assigned OTUs.

### Statistical analysis

To assess the statistical significance of differences between the two extraction methods, independent t tests (or Mann‒Whitney U tests in the case of nonnormal distribution) were performed to compare the DNA yield and quality between the QIAamp DNA FFPE Tissue Kit and the QIAamp DNA FFPE Advanced Kit. The comparison of DNA concentrations measured by spectrophotometry (NanoDrop 2000) and fluorometry (Qubit dsDNA Broad-Range assay) was also carried out via correlation analysis (Pearson or Spearman, depending on the data distribution). A *p* value of < 0.05 was considered to indicate statistical significance. All data analysis was performed via statistical software (GraphPad Prism 10.4.1).

## Results

### Optimization of the number of tissue sections required to achieve a high DNA yield

The number and quality of tissue sections used for DNA extraction are critical determinants of yield and integrity. Both inadequate and excessive sectioning can result in suboptimal DNA recovery. Additionally, factors such as the tissue block surface area and section thickness directly influence the quantity and quality of the extracted DNA. To improve DNA integrity, we avoided the use of surface-exposed sections, which are more susceptible to oxidative damage. Instead, deeper sections were selected to ensure better preservation. Furthermore, enriching the selected sections for target cells, such as tumor cells, is essential for obtaining high-quality, relevant genomic material^9^. The manufacturer’s protocol for the standard extraction kit recommends the use of eight sections of 5–10 µm thickness. Despite adhering to these guidelines, we observed insufficient yield. Consequently, we optimized the protocol to include 4–6 sections with a thickness of 10 µm, adjusting the number of sections based on the area of the tissue core which ranged between 5–30 mm^2^. For smaller cores, additional sections were included to meet the DNA input requirements (Table 1). To minimize paraffin contamination, the excess paraffin surrounding the tissue core was carefully trimmed away via a sterile scalpel and tweezers, taking care not to disturb the tissue itself. For the advanced extraction kit, we experiment with both fewer and greater numbers of tissue sections. Through this optimization process, we established that high-quality DNA yield could be consistently achieved using only 4–5 sections at a thickness of 10 µm, indicating improved extraction efficiency due to our optimized protocols (Table 2). These findings highlight the importance of tailoring extraction protocols to the specific characteristics of the tissue and the extraction method employed to maximize both the DNA yield and quality.

**Table 1:**
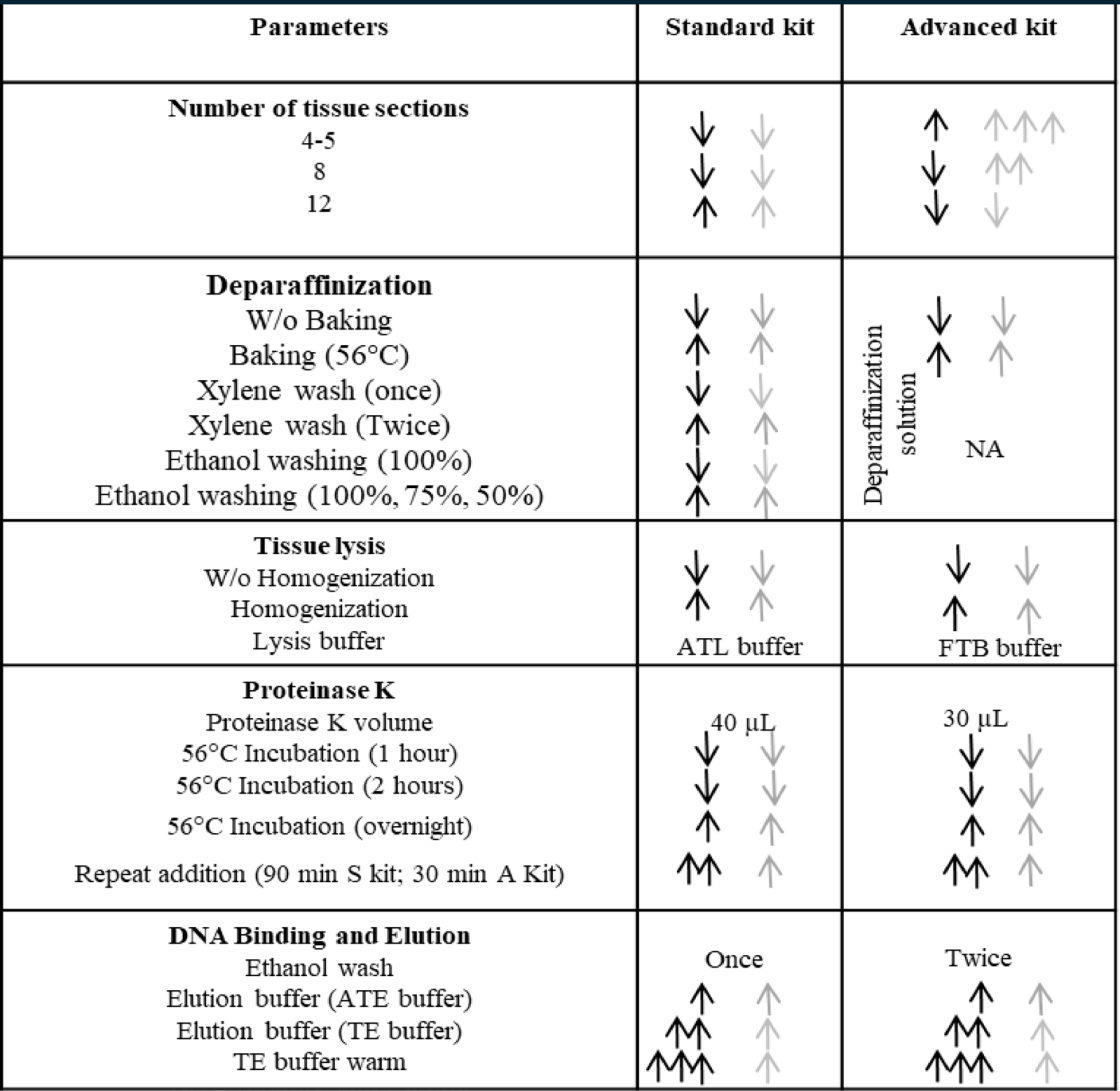
Table showing comparative differences in DNA yield (dark shaded arrows) and purity (light shaded arrows) before and after modified protocols.

**Table 2:**
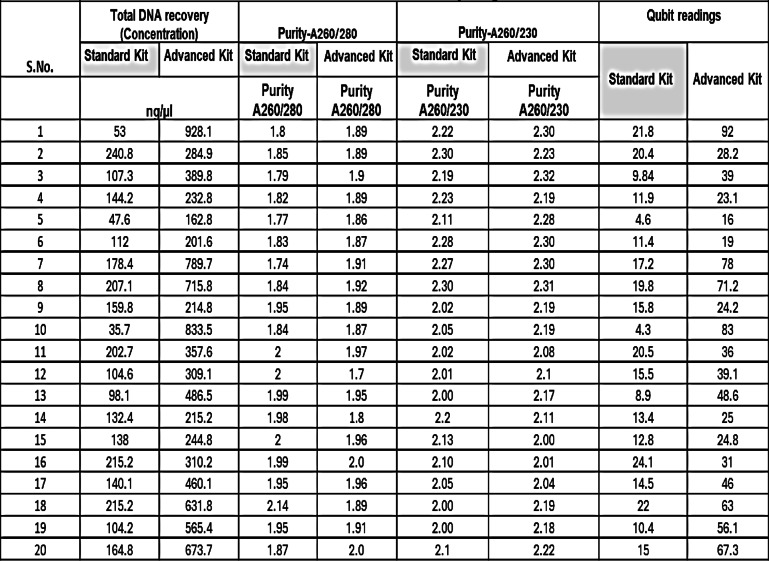
Evaluation of the effects of protocol modifications on DNA yield and purity via standard and advanced extraction kits.

### Incorporating pre-baking of FFPE tissue scrolls enhances paraffin removal and improves DNA yield and quality

We observed that baking the tissue scrolls at 56 °C is a very useful step that helps improve the deparaffinization effectiveness of DNA isolation. The incorporation of a baking step at 56 °C prior to deparaffinization significantly improved the efficiency of paraffin removal and enhanced DNA isolation, which is consistent with previous findings ^10^. Initially, following the manufacturer’s instructions, DNA extraction was performed without baking. However, we observed that in several samples, excess paraffin hindered efficient DNA recovery, resulting in suboptimal yields. To address this, tissue scrolls were baked in closed 1.5 mL Eppendorf tubes at 56 °C for 1–1.5 h prior to deparaffinization. This step facilitated melting of the paraffin, which could then be more effectively removed during the subsequent extraction process. Without baking, paraffin frequently adheres to the tube walls, compromising the efficiency of deparaffinization and downstream isolation. By softening the paraffin and reducing its adherence, the baking step improved both the yield and quality of the extracted DNA. This modification was successfully applied to both standard and advanced extraction protocols.

### Optimization of deparaffinization enhances DNA yield and quality

Deparaffinization is a crucial step in extracting DNA from formalin-fixed, paraffin-embedded (FFPE) tissue samples. The process removes paraffin wax, which interferes with the efficient release and purification of DNA. In the standard extraction kit, xylene is recommended as a mandatory deparaffinizing agent prior to DNA isolation. While widely used, xylene is a toxic compound and has been reported to adversely affect DNA quality ^11^. Following the manufacturer’s instructions, we initially applied xylene treatment with vortexing, followed by a 100% ethanol wash, centrifugation, and incubation at 37 °C to remove residual ethanol and paraffin. However, this protocol results in suboptimal DNA yield and quality. To improve outcomes, we optimized the deparaffinization process by incorporating warm xylene and washing with xylene (1 ml) twice, which significantly enhanced paraffin removal. Additionally, we modified the ethanol washing steps, like the protocols used in immunohistochemistry. Rather than using only 100% ethanol, we included sequential washes with 75% and 50% ethanol (1 min in 100%, 3 min in 75%, and 5 min in 50%), which substantially improved the DNA yield and purity (Table 1).

**Fig. 1:**
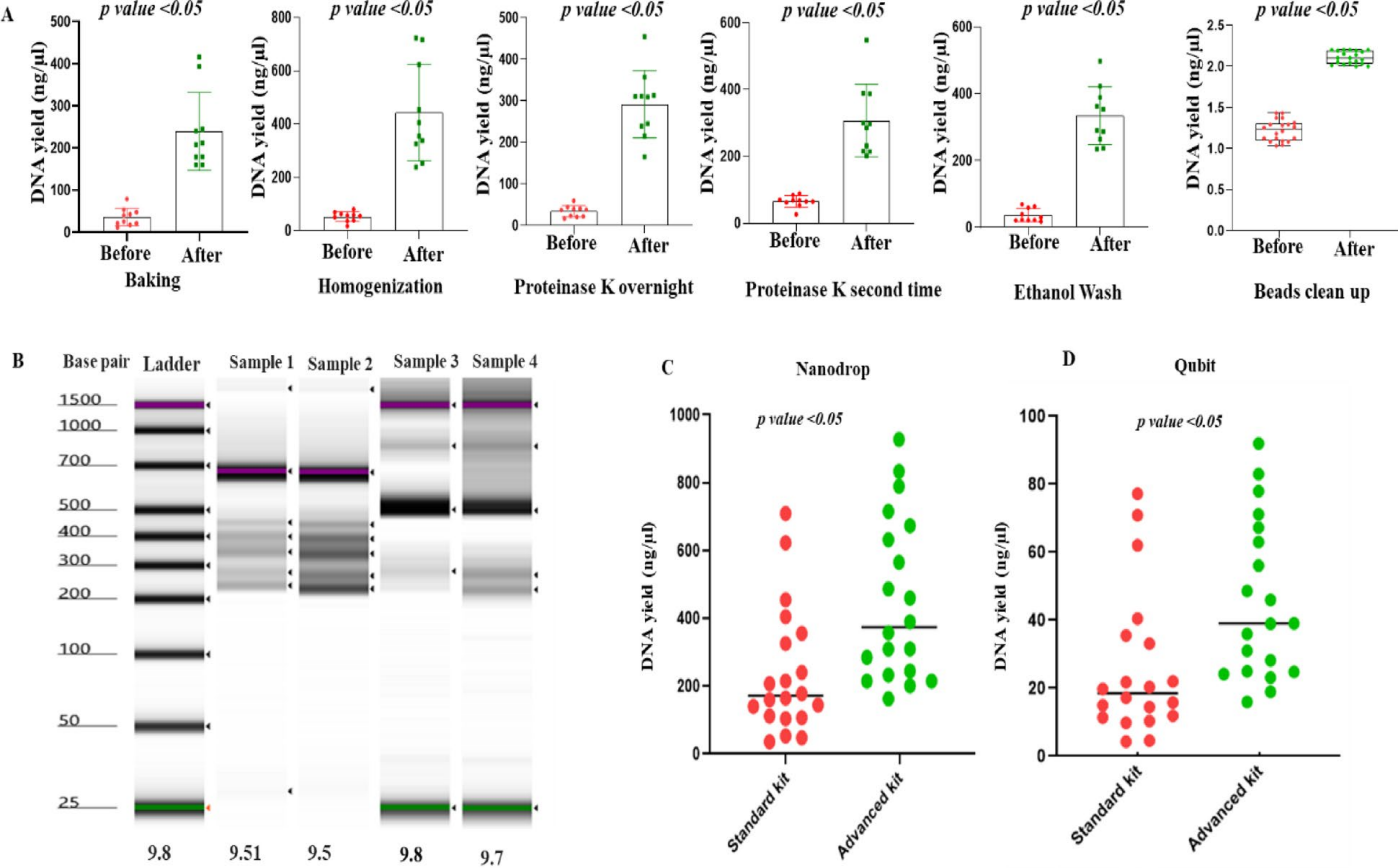
Evaluation of DNA yield and integrity using the advanced extraction kit under tested parameters. (**A**) Effect of various tested parameters on DNA yield from samples (N = 10) as measured by Nanodrop analysis. Dot plots compare the DNA yield obtained using the advanced kit with “before and after” protocol modifications described in Table 1 (**B**) Analysis of DNA integrity from samples isolated using the advanced kit and optimized protocol, assessed with the Tape Station. Image depicting DNA integrity number. (**C** and **D**) Quantitative comparisons via Nanodrop and Qubit measurements confirmed that the DNA yield from samples (N = 20) was significantly greater with the advanced kit than with the standard kit even after optimization. Statistical analysis was performed using Graph pad prism software and using Wilcoxon t-tests showing p-values < 0.05 for all the comparisons. *P* values that are less or equal to 0.05 were considered significant.

In contrast, the advanced extraction kit utilizes a proprietary deparaffinization solution that eliminates the need for xylene and ethanol. According to the kit protocol, 300 µl of the solution was added to the tissue sections, which were then vortexed and incubated at 56 °C for 3 min. While effective in part, our initial results revealed suboptimal DNA yield and integrity. We therefore optimized the incubation period to 5, 8, 10, and 20 min and found that extending the incubation time to 10 min followed by vortexing yielded significantly improved deparaffinization and DNA recovery (Fig. 1A). These protocol optimizations for both standard and advanced kits highlight the importance of tailoring deparaffinization conditions to maximize DNA extraction efficiency from FFPE tissues. Another key optimization using the advanced kit involved adjusting the volume of the deparaffinization solution when processing fewer tissue sections. Gradually increasing the solution volume from 300 µl to 400 µl led to improved DNA yield. However, owing to the limited capacity of the extraction tubes, it was necessary to balance the number of sections with the volume of the deparaffinization reagent to maximize efficiency. This optimization proved particularly valuable when working with small or limited clinical samples, where achieving high DNA yield may be challenging, but maintaining high purity is essential for downstream applications. Overall, our findings highlight the need for tailored deparaffinization strategies adapted to both the extraction method and the specific characteristics of FFPE tissues to enhance DNA isolation outcomes.

### Optimizing tissue homogenization to improve DNA yield and quality from FFPE samples

Effective lysis of formalin-fixed, paraffin-embedded (FFPE) tissue remains a significant challenge because of the dense and cross-linked nature of the samples. To enhance tissue disruption, we incorporated a homogenization step prior to lysis, which was not recommended in the instruction manual of the kits. The tissue samples were homogenized via a rotor–stator homogenizer, beginning at low speed and increasing to high speed for 2–3 s. This brief but targeted homogenization markedly improved tissue breakdown, thereby enhancing access of the lysis buffer to the cellular material (Table 1). As a result, we observed a substantial increase in both DNA yield and quality (Fig. 1A). Although occasional frothing was noted during homogenization, it was mitigated by the careful addition of RNase-free water, which did not adversely affect DNA recovery. The samples were visually inspected to ensure that no residual tissue clumps remained, as these clumps could impede subsequent steps. Proper homogenization allowed for more uniform tissue disruption and improved the efficiency of proteinase K digestion, ultimately supporting the extraction of higher-quality DNA suitable for downstream applications.

### Optimization of proteinase K treatment enhances DNA yield and quality

Proteinase K treatment is a critical step in DNA extraction from FFPE samples, as it degrades proteins that may interfere with downstream applications, thereby improving DNA purity and integrity ^12^. In this study, we optimized both the concentration and incubation conditions for Proteinase K to increase extraction efficiency. The standard and advanced kits recommend proteinase K digestion at 56 °C for only 1 h; however, we found that these conditions did not consistently yield optimal DNA across all samples especially with limited tissue. To address this, we tested various enzyme concentrations and digestion times. Increasing the proteinase K concentration from 20–40 µl from a stock of 20 mg/mL and extending the incubation period to overnight at 56 °C significantly improved lysis and DNA recovery (Table 1).

Therefore, for the standard kit, the optimized protocol included overnight digestion with 2.66 mg/ml proteinase K at 56 °C, followed by an additional 90 min of digestion at 1.76 mg/ml at 90 °C. For the advanced kit, a similar overnight proteinase K digestion was performed using 1.2 mg/ml at 56 °C. This was followed by incubation at 90 °C for 1 h. After removal of the upper blue phase of the deparaffinization solution, 610 µg/ml proteinase K was added to the sample, and the incubation at 65 °C was extended from 15 to 30  min with shaking at 450 rpm. These adjustments consistently increased the DNA yield and quality, making the extracted DNA more suitable for downstream applications, as shown in Fig. 1A and Table 1 using Advanced kit. Our findings underscore the importance of tailoring the enzyme concentration and incubation parameters to the sample type and extraction method for improved performance.

### Optimization of washing and elution steps enhances DNA purity and recovery

Both standard and advanced extraction kits employ silica-based spin columns for DNA binding, a widely used approach that enables effective capture of nucleic acids during the extraction process. While the manufacturer’s protocols for washing and elution were followed as a baseline, several modifications were introduced to increase the DNA yield and purity. To improve the removal of residual salts, proteins, and other contaminants, we incorporated an additional ethanol washing steps beyond the single wash recommended in the protocol. This additional ethanol washing steps significantly improved DNA purity by further eliminating impurities that can interfere with downstream applications such as PCR, qPCR, and sequencing. Ethanol washing plays a critical role in maintaining the integrity of extracted DNA by ensuring thorough purification while the remaining DNA is bound to the silica matrix. For the elution step, both kits originally recommended the use of proprietary ATE buffer. However, we substituted this with laboratory-prepared TE buffer (10 mM Tris, 1 mM EDTA, pH 8.0), which provided comparable or improved results (Table 1). Prior to elution, the TE buffer was prewarmed to increase the solubility and release of bound DNA from the column. Elution was carried out by applying prewarmed buffer to the column, followed by a 5-min incubation at room temperature and subsequent centrifugation. To maximize DNA recovery, a second elution was performed using an additional volume of prewarmed TE buffer. This second elution effectively recovered residual DNA that remained bound after the first elution, thereby increasing the overall DNA yield without compromising quality (Table 1). To further evaluate the performance of each protocol, DNA integrity was assessed via the Agilent 4200 Tape Station system. The Tape Station results revealed that DNA extracted via the advanced kit consistently displayed higher-molecular-weight fragments and sharper bands, indicating better preservation of DNA integrity and suitability for the use of samples for sequencing downstream applications (Fig. 1B). Quantitative comparisons via Nanodrop and Qubit measurements confirmed that the DNA yield was significantly greater with the advanced kit than with the standard kit (Fig. 1C–D). These findings aligned with the quantitative results obtained from the Nanodrop and Qubit assays, reinforcing the superior performance of the advanced extraction method. Collectively, these optimized washing and elution steps, along with quality assessments by Tape Station, contributed significantly to the reproducibility, integrity, and reliability of DNA extraction from FFPE samples across both standard and advanced protocols (Tables 1 and 2).

### Improving purity DNA via magnetic bead-based clean-up

In few DNA extractions using either standard or advanced extraction kits, while the DNA yield was within an acceptable range, the purity, specifically the A260/A230 ratio, fell below the standard threshold of 1.8–2.0, suggesting the presence of residual contaminants such as salts, proteins, or organic compounds (Table 2). These impurities can compromise sensitive downstream applications such as next-generation sequencing or quantitative PCR. To address this, we implemented a clean-up step using AmpPure magnetic beads, a widely adopted method for improving DNA purity without significantly compromising yield. The clean-up procedure involved binding the DNA to AmpPure beads, followed by two successive washes with 100% ethanol to remove copurified contaminants. This washing strategy ensures the efficient elimination of interfering substances while preserving the integrity of DNA. Following the washes, the DNA was eluted in prewarmed TE buffer (pH 8.0), which facilitated the dissolution of any residual impurities and improved the elution efficiency. Although slight reduction in DNA yield was observed post clean-up, the trade-off was justified by the substantial improvement in purity, particularly an increase in the A260/A230 ratio to within or above the acceptable range (Fig. 1A). This enhancement was critical for samples intended for high-resolution and precision-dependent applications. Quantitative analysis further supported the superior performance of the advanced kit. The results of the Mann‒Whitney U test revealed that DNA quantity was significantly greater with the advanced kit, as measured by both Nanodrop (median: 373.7 ng vs. 109.65 ng, p = 0.00025) and Qubit (median: 37.5 ng vs. 19.75 ng, p = 0.0357) (Table 3) methods, indicating improved yield and consistency across replicates (Table 3). Overall, the bead-based purification method effectively increased DNA purity and proved especially useful when working with samples that initially exhibited high yields but suboptimal purities. This approach provides a reliable and scalable strategy for improving DNA quality in FFPE tissue extractions.

**Table 3:**
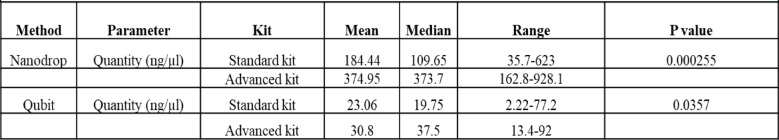
Mann‒Whitney U test to compare the readings of standard and advanced kits.

### Assessment of DNA quality and its suitability for downstream applications

**Fig. 2:**
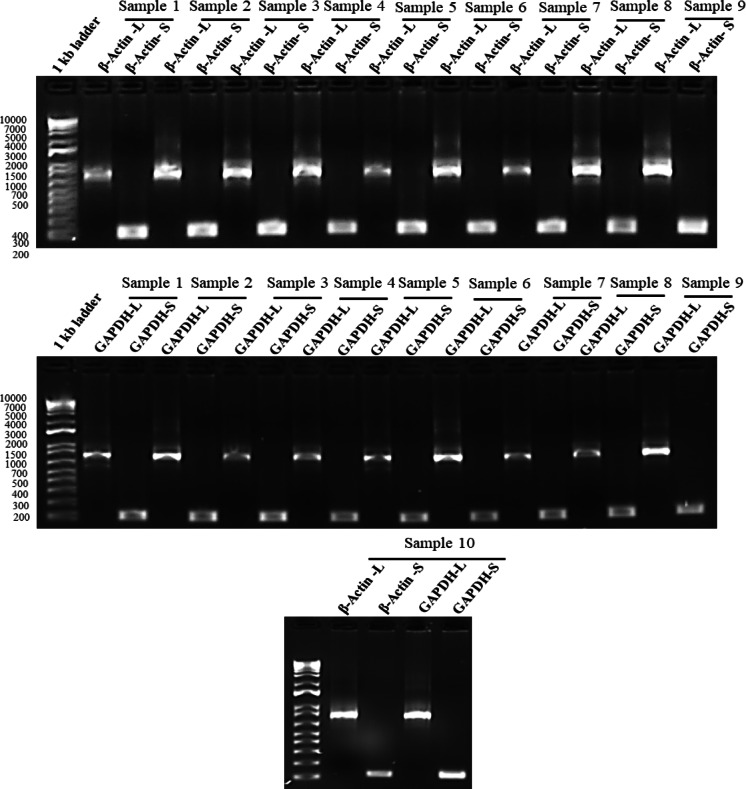
Length-dependent PCR demonstrates high-quality DNA from FFPE tissue. DNA from the optimized protocol was amplified for β-Actin and GAPDH using primers generating short (~ 200 bp) and long (> 1000 bp) fragments.

To evaluate the functional integrity of DNA extracted using our optimized protocol, we performed length-dependent PCR assays targeting a single-copy genomic locus. Amplicons ranging from ~ 200 bp to > 1000 bp were amplified using equal DNA input to assess the DNA’s ability to support both short- and long-fragment amplification (primer sequences are provided in Supplementary Table S2). Our results show that DNA extracted with the optimized protocol successfully amplified both short and long fragments, demonstrating that the DNA is of high functional quality (Fig. 2). This indicates that the protocol preserves DNA integrity sufficiently to support downstream applications that require longer templates, beyond what is detectable by standard short-amplicon PCR. These findings provide direct evidence that the optimized extraction method yields DNA of superior functional integrity, suitable for diverse downstream molecular workflows, and directly addresses concerns regarding the performance of FFPE-derived DNA in longer-range PCR applications.

**Fig. 3:**
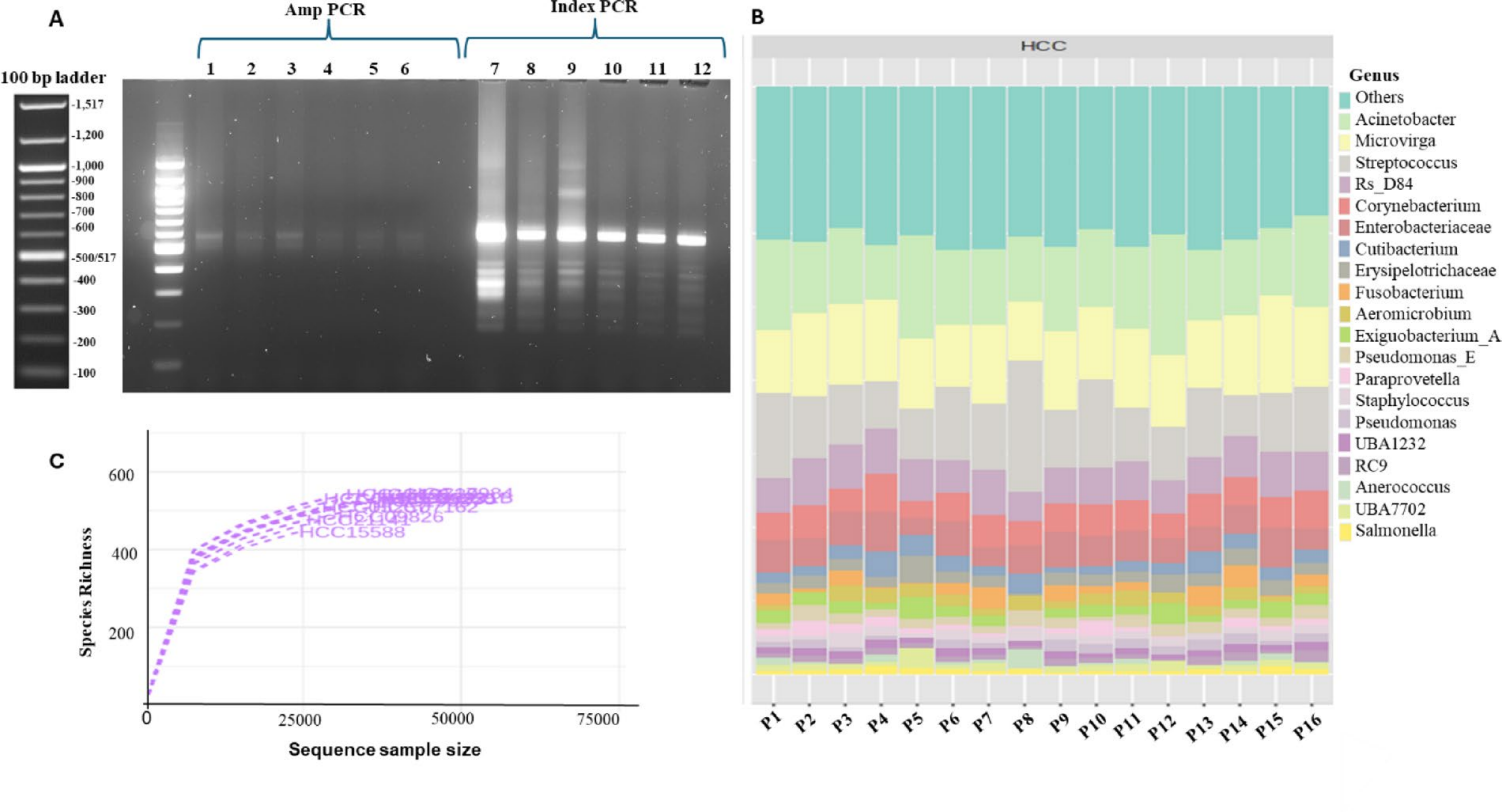
Validation of library preparation and microbial diversity analysis from FFPE-derived DNA of HCC patients using the advanced kit and modified protocol. (**A**) Agarose gel electrophoresis (2%) confirming successful amplification of library components from FFPE-derived DNA samples (N = 12). Lane 1: 100 bp DNA ladder; Lanes 2–8: Amplicon PCR products; Lanes 9–14: Index PCR products. (**B**) Genus-level bacterial abundance profile in libraries prepared from FFPE tissue samples (N = 16) of HCC patients. (**C**) Rarefaction curves illustrating species richness across HCC FFPE samples, indicating sequencing depth and microbial diversity. The statistical tests used for analysis were Welch T-test/ANOVA using online tool Microbiome Analyst to generate genus-level diversity and Rarefaction curves.

Further, we performed 16S rRNA gene-based metagenomic sequencing using Illumina MiSeq platform and Illumina-compatible primers targeting the V3–V4 region. High-quality DNA was successfully used to construct sequencing libraries, which included the generation of distinct amplicon and index PCR products, as visualized by agarose gel electrophoresis (Fig. 3A). Clear, well-defined bands confirmed efficient amplification and the absence of nonspecific products, indicating that the isolated DNA was of high integrity and suitable for library preparation.

Sequencing on the Illumina MiSeq platform generated high-depth, high-quality reads, with consistent amplicon sizes corresponding to the expected V3–V4 region. Subsequent bioinformatic analysis produced robust clustering into operational taxonomic units (OTUs) and accurate taxonomic classification using the SILVA database. The resulting microbial diversity and taxonomic profiles demonstrated reliable and reproducible sequencing performance, thereby confirming that the DNA extracted using the optimized protocol possessed sufficient purity, integrity, and representativeness for comprehensive microbiome and other downstream genomic applications (Fig. 3B–C).

## Discussion

Formalin-fixed paraffin-embedded (FFPE) tissue samples constitute an indispensable component of clinical and research biobanks worldwide, serving as a critical bridge between archived biospecimens and contemporary molecular analyses. Despite their utility, FFPE tissues pose formidable barriers to high-quality DNA extraction owing to formalin-induced cross-linking, fragmentation, and chemical modifications. These challenges often translate into suboptimal yields and compromised nucleic acid integrity, significantly affecting the reliability and reproducibility of downstream applications such as PCR, genotyping, and next-generation sequencing.

**Fig. 4:**
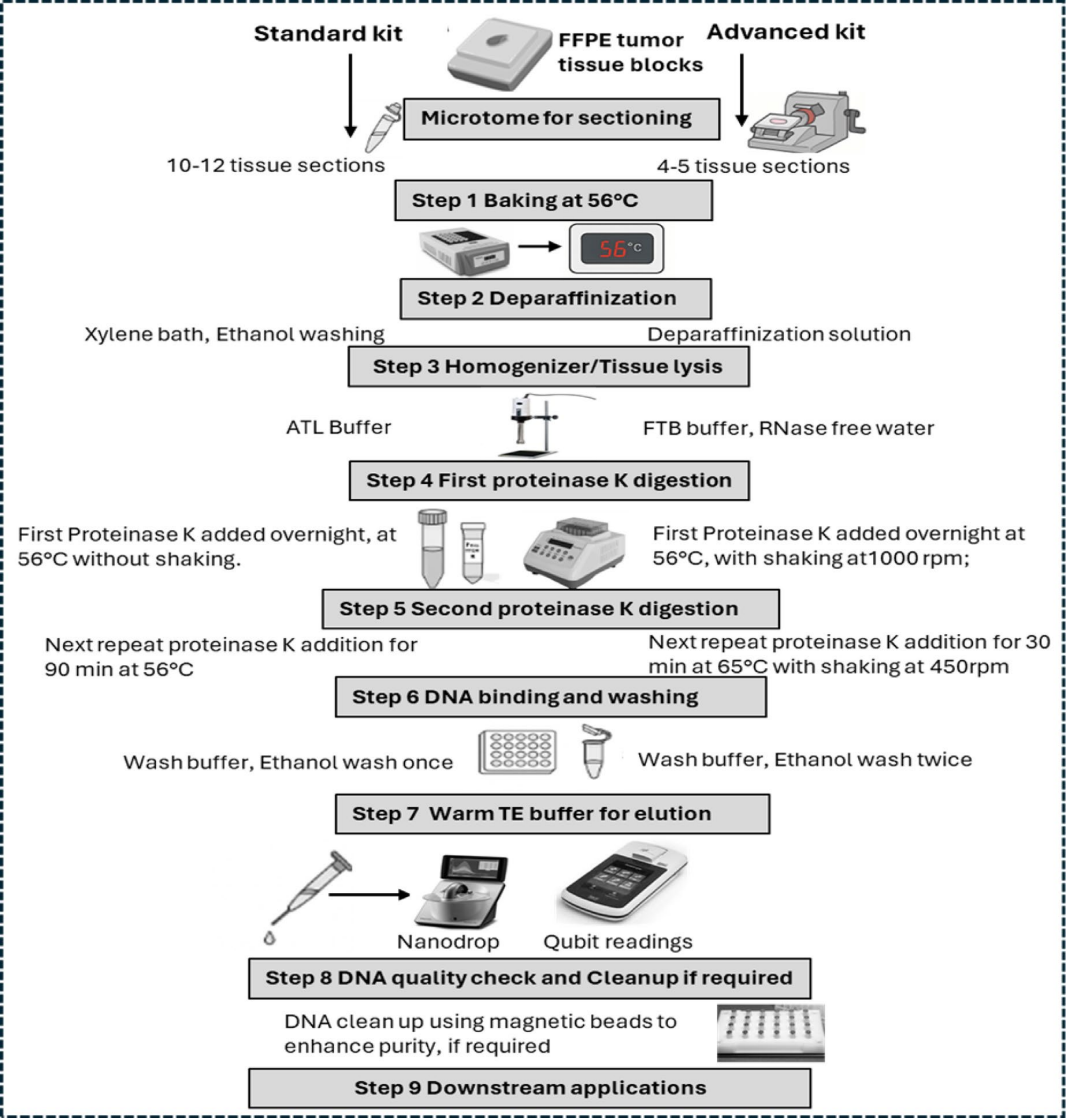
Workflow describing the main steps involved in genomic DNA isolation using both standard and advanced kit to ensure high-purity DNA is suitable for downstream applications.

Our study provides a comprehensive and pragmatic roadmap for improving DNA isolation from FFPE tissues through protocol refinements applied to both the standard Qiagen FFPE DNA kit and the advanced version. By systematically optimizing each step from deparaffinization to proteinase K digestion and final elution, we demonstrated a substantial improvement in both DNA yield and purity across a wide range of clinical and environmental FFPE samples (Fig. 4). Notably, the advanced extraction kit performed better than the standard kit when used with our modified protocols, yielding higher-quality DNA and improved consistency.

A pivotal enhancement in our approach was the prebaking of scrolls, which significantly improved paraffin removal and led to increased DNA yield and purity. In standard kits, traditional xylene-based deparaffinization, although widely used, is often inefficient and toxic. Our data show that deparaffinization with warm xylene and the introduction of a second xylene wash improved paraffin clearance, yielding better results than did the manufacturer-recommended single-step approach. Moreover, ethanol gradient washes (100%, 75%, 50%) further facilitated the removal of residual paraffin and improved tissue permeability during lysis. In the case of the advanced kit, we found that increasing the volume of deparaffinization solution, especially when fewer tissue sections were used, substantially enhanced DNA recovery. However, because tube volume imposes physical limitations, a balance between tissue input and reagent volume must be achieved to optimize recovery. This consideration is particularly crucial when working with rare or limited clinical specimens, where maximizing DNA integrity is often prioritized over yield alone. Crucially, the advanced kit requires fewer tissue sections. (4–5 vs. 10–12 in the standard kit) without compromising DNA yield or purity, provided that the deparaffinization buffer volume is appropriately increased. This finding highlights a key advantage in conserving valuable archival material, especially in cases where only limited FFPE tissue is available. Our results emphasize the importance of calibrating the number of sections on the basis of the tissue core size to avoid saturation effects or insufficient material, both of which negatively affect extraction efficiency.

Another critical modification involves the incorporation of tissue homogenization prior to lysis. We observed a substantial increase in DNA yield and integrity following mechanical homogenization of tissue sections prior to enzymatic digestion. This mechanical disruption ensured complete tissue breakdown, enhancing the penetration and efficiency of the lysis buffer. While traditional protocols rely solely on chemical lysis, the inclusion of high-speed homogenization ensures more complete disruption of the cellular and extracellular matrices, enhancing the penetration of proteinase K and facilitating thorough digestion. These improvements were reflected in both higher yields and better purity ratios, as mechanical disruption reduced the prevalence of protein‒DNA complexes that could otherwise inhibit binding to silica membranes. When optimized proteinase K digestion was combined with higher concentrations (40 mg/ml) and incubation was extended overnight, we observed markedly improved DNA recovery. Furthermore, dual-stage digestion (overnight followed by shorter secondary incubations) proved especially effective in minimizing residual proteins and maximizing DNA release.

Our findings also highlight the importance of refining the DNA binding and elution steps. Doubling ethanol washing steps improved the removal of residual salts and organic impurities, leading to increased A260/A230 ratios. Additionally, substituting the proprietary ATE elution buffer with laboratory-prepared TE buffer (pH 8.0) and prewarming it prior to elution significantly improved the release of DNA from the silica columns. Performing a second elution further maximized recovery, especially from low-yield samples. In samples with high yield but suboptimal purity, postextraction clean-up using AmpPure magnetic beads was highly effective. Despite minor losses in DNA quantity, the improvement in purity, particularly in A260/A230 ratios, was substantial and essential for downstream applications requiring high-fidelity input, such as sequencing and methylation analysis.

Collectively, our optimized protocol demonstrates that small, targeted modifications to existing commercial kits can dramatically increase the quality and quantity of DNA obtained from FFPE tissues. These refinements are broadly applicable and do not require expensive instrumentation or proprietary reagents, making them accessible to a wide range of laboratories. Notably, they offer a reliable approach for obtaining high-quality DNA from challenging sources such as degraded, limited, or archival tissue samples, conditions frequently encountered in retrospective clinical research and precision medicine efforts. Compared with the standard protocol, advanced integrated optimization methods, including nontoxic deparaffinization, minimal sample input, simplified processing, and enhanced wash steps, produce consistently higher-quality DNA with greater ease and reproducibility (Fig. 4).

In conclusion, these improvements are especially valuable for clinical laboratories and research facilities handling large-scale molecular profiling of FFPE cohorts, including retrospective cancer studies and population-scale genomic surveillance. Certain steps, such as overnight incubations or extended digestion, may increase workload and prolong turnaround time for analyses. However, this optimized protocol offers a clear advantage when the objective is to maximize DNA yield and purity for research and comprehensive genomic analyses using limited FFPE tissue input. A faster version of the protocol can also be implemented by omitting overnight enzymatic incubations when sample quantity is sufficient. Because formalin fixation can lead to deaminated-cytosine artifacts that may result in false-positive variant calls during mutational analyses, incorporating UNG-based uracil digestion during DNA isolation can help minimize false-positive single-nucleotide variant reports. As the demand for reliable nucleic acid extraction from FFPE tissues continues to grow, fueled by advances in single-cell genomics, spatial transcriptomics, and multiomics, the need for adaptable and high-efficiency protocols becomes increasingly important. This study presents a robust and reproducible method that connects traditional biospecimen preservation practices with the stringent quality standards of modern genomics, enabling more precise and comprehensive molecular analysis of archived samples.

## Supplementary Information

Below is the link to the electronic supplementary material.

Supplementary Material 1

## Data Availability

This manuscript contains metagenomic sequence data from tumor tissues. The datasets generated and/or analyzed during the current study are available are archived at NCBI Sequence Read Archive (SRA) under the BioProject accession number PRJNA1297695 (http://www.ncbi.nlm.nih.gov/bioproject/1297695).
