# Supplementary material for: Enhanced recovery of high-quality DNA from limited FFPE tissue for advancing cancer genomics
Source: Sci Rep. 2026 May 23;16:23521. doi: 10.1038/s41598-026-51594-9 (PMC13415559; doi:10.1038/s41598-026-51594-9)
Supplement: Supplementary file 1 — Supplementary Material 1 [file 41598_2026_51594_MOESM1_ESM.docx]

**Supplementary Results**


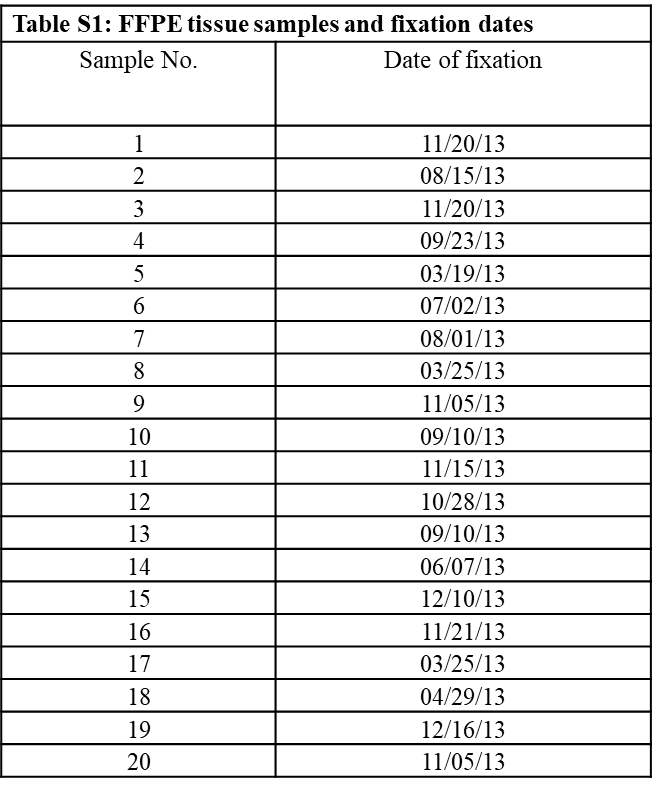


**Supplementary Table S1: FFPE tissue samples and fixation dates.** List of the tissue samples used in this study, including the sample number and the corresponding date of formalin fixation. This information provides context for sample age, which is relevant for assessing DNA integrity and suitability for downstream applications.


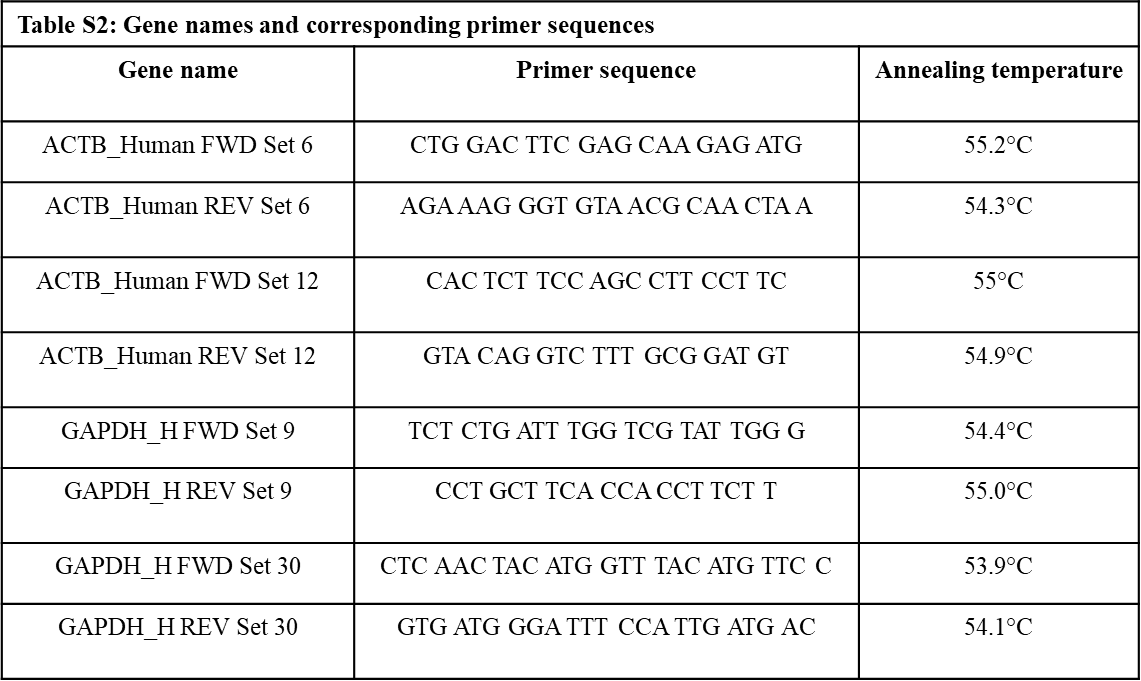


**Supplementary Table S2**. Gene names and corresponding primer sequences. This table lists the genes analyzed in this study along with the sequences of the forward and reverse primers used for PCR amplification.
